# A review on gastric diverticulum

**DOI:** 10.1186/1749-7922-7-1

**Published:** 2012-01-18

**Authors:** Farhan Rashid, Ahmed Aber, Syed Y Iftikhar

**Affiliations:** 1 Department of Upper GI Surgery, Royal Derby Hospital, Graduate Entry Medical School, Derby, University of Nottingham, UK; 2 Department of Surgery, Luton and Dunstable Hospital, Luton, UK

## Abstract

The gastric fundal diverticulae are rare. They can present with variable symptoms. We are enclosing a literature review on gastric fundal diverticulum. Lessons have emerged which may help in the management of this rare condition in future.

## Introduction

Gastric diverticulum (GD) is an outpouching of the gastric wall. GDs are rare and they are commonly detected incidentally during routine diagnostic testing. Prevalence ranges from 0.04% in contrast study radiographs and 0.01% - 0. 11% at oesophagogastrodeudenum (OGD) [1,2]. The incidence of gastric diverticulum is equally distributed between males and females and typically may present in the fifth and sixth decades. However it is worth mentioning that it may present in patients as young as 9 years old [3].

The lack of exact pathogonomic symptoms and the vague long history of presenting complaints that can range from dyspepsia to major upper gastrointestinal (GI) bleed make this condition a diagnostic challenge.

We conducted a literature search using the "Pubmed" search engine. The following terms "gastric diverticulum" and "Stomach diverticulum" were used to identify the appropriate papers.

In this review, our emphasis is to highlight on the presentation, the pathophysiology, investigations and different management options for this condition.

### Presentation of gastric diverticulum

Symptoms of GD vary and can imitate those of other common disorders. It is important to note that most GD are asymptomatic but may present with a vague sensation of fullness or discomfort in the upper abdomen. Presenting complaint might also be the result of a major complication of GD. This includes acute upper gastrointestinal bleed or perforation [1,2] (Table 1).

**Table 1:** GD presenting symptoms, diagnostic investigations and management.

Symptoms	Investigation	Management	Refs
Incidental finding on CT scan	Upper GI contrast study/CT with oral contrast	None	18, 19
Upper GI bleed	OGD	OGD & Adrenaline injection	22, 23
Upper abdominal pain, reflux, bloating	CT with contrast & OGD	Laparoscopic surgical resection	1,5, 29, 30, 31
Upper abdominal pain and anorexia	OGD	PPI	5, 9
Upper abdominal pain	Upper GI contrast study	Exploratory laparotomy plus diverticulectomy	5

### Patho-physiology

GD in general is a rare condition; It is found in 0.02% (6/29 900) of autopsy studies and in 0.04% (165/380 000) of upper gastrointestional studies [1,3,4]. Meeroff *et al *reported a prevalence of 0.1-2.6% in an autopsy series [4].

Seventy-five percent of true gastric diverticula were located in the posterior wall of the fundus of the stomach, 2 cm below the oesophagastric junction and 3 cm from the lesser curve. False diverticula were either traction or pulsion and associated with inflammation, other diseases, or both. Diverticula were usually less than 4 cm in size (range, 3 cm to 11 cm) [5,6].

In the literature review we did identify a proposed hypothesis explaining the pathophysiology of this condition. This hypothesis classifies GD cases into congenital and acquired types, with congenital types being more common [5-8]. Based on a review of embryogenesis it had been suggested how a gastric diverticulum can be located within the retroperitoneal space in an attempt to explain the commonest type to GD.

In the period between the 20th and 50th day of gestation, the stomach is transformed from a fusiform swelling of the foregut into its adult form. At this time, there is a 90° rotation of the stomach, which carries with it the duodenum, the pancreas, and the dorsal mesentery. The posterior body wall and dorsal mesentery then fuse encapsulating the pancreas within the retroperitoneum and establishing its adult form [9].

A diverticulum of the posterior wall of the gastric fundus hypothetically could herniate through an area of dorsal mesentery before its fusion with the left posterior body wall. Initially, the diverticulum would lie superior to the pancreas. With further extension, the diverticulum could project posterior to the pancreas.

Acquired gastric diverticula in contrast are pseudodiverticula, less common and typically located in the antrum. They usually present with a background history of other gastrointestinal pathology, such as peptic ulcer disease, malignancy, pancreatitis, or gastric outlet obstruction. Gastric diverticula had been reported following surgical procedures on the stomach, including Roux-en-Y gastric bypass [4,10,11].

### Investigations

Accurate diagnosis is essential given the risk for severe complications, including bleeding and perforation, as well as the association with ectopic mucosa and potential for malignant transformation [12]. The condition can be diagnosed by radiological or endoscopic examinations.

This is usually accomplished with upper gastrointestinal contrast radiographic study (UGI) or oesophagogastrodudenoscopy (OGD). These are the most reliable diagnostic tests but reports in the literature confirm that they can give false negative results [13,14]; especially for a diverticulum with a narrow neck that precludes entry of the contrast or scope. It is stated that the GD is best identified during UGI study using a right, anterior oblique view with the patient in a supine, slightly left lateral decubitus and Trendelenburg position [13-16]. In a large review, Palmer [13] reported that 14 of 262 (5%) GDs are missed during UGI study. Other reports support the use of OGD [10,17] for diagnosis. Distension of the diverticulum by the scope may mimic the patient's symptoms and this maneuver may indicate which patients would benefit from resection [10]. Other reports suggest that computer tomography scanning may be effective; however, the accuracy of this imaging modality is not widely accepted because of the possible misdiagnosis [18,19].

### Management

There is no specific treatment plan for an asymptomatic diverticulum [9,20]. The appropriate management for a symptomatic GD depends mainly on the severity of the presenting complaints.

### Medical and non surgical therapy

Protein pump inhibitors therapy for few weeks is reported to resolve the symptoms in proven cases of GD [9]. However it is important to note that this does not resolve the underlying pathology and some studies report that patients presented again with refractory symptoms of dyspepsia and worsening epigastric pain that did not settle with either protein pump inhibitors or histamine receptor blockers [21].

There are also reports in the literature of successful endoscopic management of cases of gastric diverticulum that presented with active upper GI bleed. None of these studies reported any further complications that warranted further surgical management [22,23].

### Surgical management

Surgical resection is recommended when the diverticulum is large, symptomatic or complicated by bleeding, perforation or malignancy. Both open and laparoscopic resection yield good results. Palmer noted that 6 of 9 patients with symptoms caused by gastric diverticulum who underwent open surgery experienced excellent outcomes [24].

Laparoscopic resection of gastric diverticulum was first described by Fine in 1998 [25]. Since then several cases using the laparoscopic surgical approach have been reported [1,26-32]. All of these cases were successfully managed by laparoscopy, with primary resection of the true gastric diverticulum.

The laparoscopic approach has been described by different authors. The most favourable approach that provides the necessary exposure is by placing the ports in a similar fashion to laparoscopic Nissen fundoplication. This includes a midline port, right upper quadrant, and 2 left upper quadrant ports. The laparoscopic dissection has been performed by either releasing the gastrocolic/gastrosplenic ligament or by mobilizing the short gastric vessels, thus gaining exposure of the superior posterior wall of the stomach. The latter is the most frequently used approach [24,25,27,28]. Because all diverticula were true and located in the gastric fundus, the most direct approach was by taking down of the short gastric vessels. Simple resection of the diverticulum with a laparoscopic cutting stapler was reported to be successful [32]

### Recent experience of dealing with gastric fundal diverticulum

**Figure 1:**
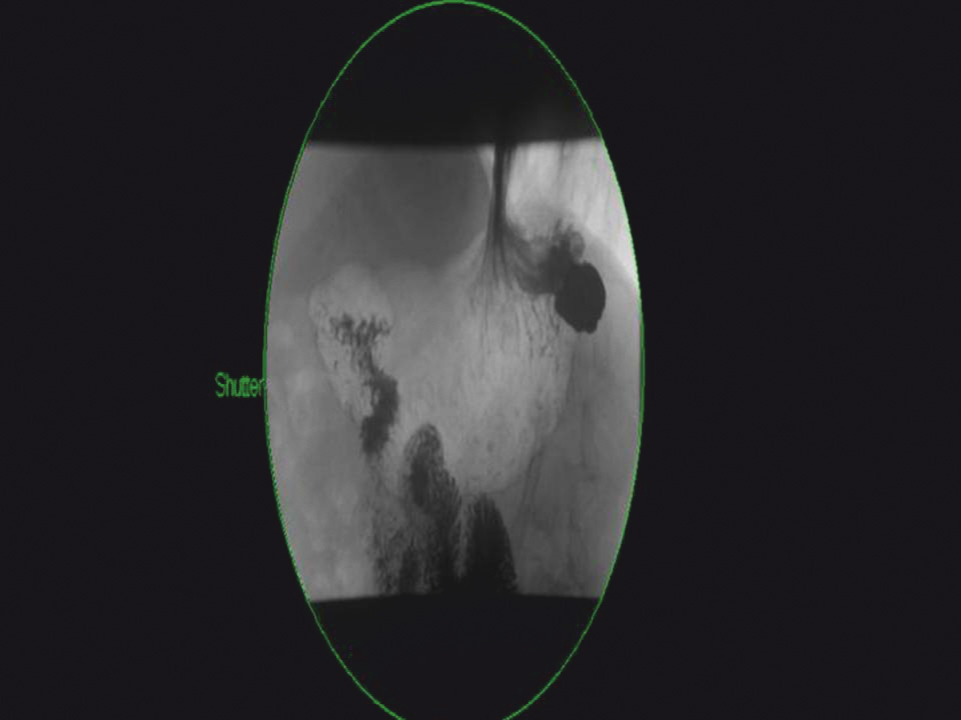
**Barium swallow study**.

A 46 year old male patient, with a 10 year history of GORD, presented with abdominal discomfort and haemoptysis. He had also felt nausea and belching with some foul smell. On examination, his abdomen was soft and non tender. He denied any weight loss and was systemically well. All investigations looking for a respiratory cause for his haemoptysis were normal. OGD revealed a gastric fundal pathology, and a small hiatus hernia. The pathology was confirmed with a barium swallow study (Figure 1).

**Figure 2:**
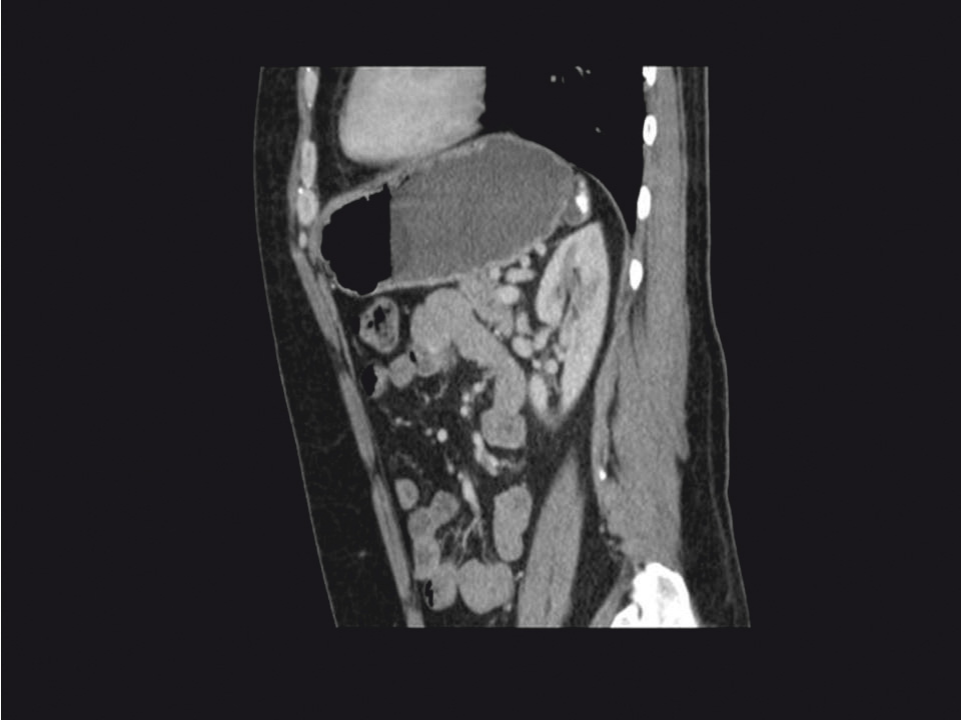
**Computed tomography**.

The computed tomography (CT) scan has shown a posterior gastric fundal diverticulum (Figure 2), containing calcified material and measuring approximately 30 mm in diameter. The patient underwent laparoscopic excision of gastric fundal diverticulum and had an uneventful recovery from the operation. The histology of the diverticulum confirmed the normal lining of the stomach. The patient remained asymptomatic on further follow up after 1 year.

## Conclusion

A high clinical index of suspicion is needed to diagnose and effectively manage patients with gastric diverticulum. This condition typically present with a long history of vague symptoms such as upper abdominal pain and dyspepsia. It does not always resolve with PPIs and can even be missed on OGD or CT scanning. A focused investigation to look for this particular condition is needed to identify it and subsequently manage it.

We particularly recommend that keep a high index of suspicion in mind especially in patients with a long history of upper abdominal pain and dyspepsia that does not resolve with PPIs and with an insignificant OGD. Although the literature does not describe a standarised approach for the management of this condition, however, we consider laparoscopic repair to be a safe and suitable procedure for this in symptomatic patients who have not responded to medical therapy.

## Consent

Written informed consent was obtained from the patient for publication of this case report and accompanying images. A copy of the written consent is available for review by the Editor-in-Chief of this journal

## Competing interests

The authors declare that they have no competing interests.

## Authors' contributions

FR and AA performed the literature search, extracted the data and wrote the manuscript. SY helped with radiological images and performed the operation. FR, AA and SYI all helped in writing different subsections of the review. All authors contributed to the manuscript, and all read and approved the final version
